# Effectiveness of introducing a 20-gauge core biopsy needle with a core trap in EUS-FNA/B for diagnosing pancreatic cancer

**DOI:** 10.1186/s12876-020-01583-7

**Published:** 2021-01-06

**Authors:** Shunsuke Watanabe, Jun Miyoshi, Masao Toki, Komei Kambayashi, Shuichi Kitada, Takeshi Nosaka, Tomoyuki Goto, Hirotaka Ota, Kazushige Ochiai, Koichi Gondo, Nobuhito Ikeuchi, Shujiro Tsuji, Kenji Nakamura, Junji Shibahara, Tadakazu Hisamatsu

**Affiliations:** 1 Department of Gastroenterology and Hepatology, Kyorin University School of Medicine, 6-20-2 Shinkawa, Mitaka-shi, Tokyo, 181-8611 Japan; 2 Department of Gastroenterology and Hepatology, Tokyo Medical University, 6-7-1 Nishishinjuku, Shinjuku-ku, Tokyo, 160-0023 Japan; 3 Department of Gastroenterology and Hepatology, Tokyo Dental College Ichikawa General Hospital, 5-11-13 Sugano, Ichikawa-shi, Chiba, 272-8513 Japan; 4 Department of Pathology, Kyorin University School of Medicine, 6-20-2 Shinkawa, Mitaka-shi, Tokyo, 181-8611 Japan

**Keywords:** Pancreatic cancer, Endoscopic ultrasound-guided fine needle aspiration/biopsy, ProCore 20-gauge needle, Diagnostic accuracy

## Abstract

**Background:**

Endoscopic ultrasound-guided fine-needle aspiration/biopsy (EUS-FNA/B) is a standard method for pathological diagnosis of pancreatic solid lesions. The EchoTip ProCore 20G® (PC20), a 20-gauge biopsy needle with a forward-bevel core trap, has been available in Japan since 2015.

**Methods:**

We compared the efficacy of the PC20 with that of the EchoTip ProCore 22G® (PC22) and Acquire 22G® (AC22) in EUS-FNA/B for diagnosing pancreatic cancer. This retrospective study included 191 patients with pancreatic cancer who underwent EUS-FNA/B using the PC20, PC22, or AC22 at our facility from April 2013 to October 2019. We investigated the patients’ clinical characteristics and the diagnostic accuracy and safety of each needle.

**Results:**

A sufficient stroke length of puncture was secured in all patients. The maximum length under EUS was shorter with the AC22 (22.1 ± 2.2 mm) than PC20 (30.6 ± 0.7 mm, *p* < 0.01) and PC22 (30.3 ± 0.8 mm, *p* < 0.01). The histological accuracy was 96.4% with the PC20 but only 58.8% with the PC22 (adjusted *p* (*p-*adj) < 0.0001) and 75.0% with the AC22 (*p-*adj = 0.06). The diagnostic accuracy of the combination of histology and cytology was 96.4% with the PC20, while it was 72.1% with the PC22 (*p-*adj < 0.0001) and 91.7% with the AC22 (*p-*adj > 0.99). One patient (0.9%) in the PC20 group developed mild pancreatitis, but no adverse events occurred with the other needles.

**Conclusions:**

The PC20 showed better diagnostic capability than the PC22. The diagnostic efficacy was similar between the PC20 and AC22. The high histological accuracy of the PC20 could be advantageous for lesions in which histological assessment is critical.

## Background

Pancreatic cancer is a highly aggressive tumor with a poor prognosis [1–5]. The age-standardized incidence rate of pancreatic cancer increased by 1.03% per year from 1973 to 2014 [6]. Changes in environmental and behavioral factors rather than genetic factors are thought to be related to the worldwide increase in pancreatic cancer [7, 8]. Differences in lifestyle (e.g., high-calorie diet and high body mass index), exposure to environmental risk factors (e.g., smoking), and accessibility to medical services including more efficient diagnostic tools might be associated with the variability in the mortality of pancreatic cancer among countries [7, 9, 10].

Pancreatic cancer is typically found as a pancreatic solid lesion. Pancreatic solid lesions include pancreatic malignant lesions such as pancreatic cancer, pancreatic neuroendocrine tumors, and metastatic pancreatic cancer as well as benign lesions such as autoimmune pancreatitis and mass-forming pancreatitis. Therefore, prompt and accurate diagnosis of pancreatic cancer is critical to determine the most appropriate therapeutic strategies. For this purpose, pathological diagnosis with minimal invasion is crucial. Endoscopic ultrasound-guided fine-needle aspiration/biopsy (EUS-FNA/B) is the standard method for sampling pathological specimens from a pancreatic solid lesion. The reported diagnostic sensitivity, specificity, and accuracy are 91.7–93.4%, 100%, and 92.3% to 94.4%, respectively [11, 12]. Various factors, including the type of puncture needle, puncture route, number of passes, tumor size, and experience of the endoscopist and cytopathologist, can impact the accuracy of diagnosis [13–15]. Several types of puncture needles with various characteristics (e.g., thickness and needle-tip shape) are commercially available [16–18]. Also, the needles are categorized into FNA needles and FNB needles and the systematic review by Li et al. [19] presented FNB are superior. The volume of a specimen obtained by core needle biopsy can depend on the thickness of the puncture needle; i.e., a needle with a larger diameter can collect a larger biopsy specimen [20]. A higher sample volume contributes to a more accurate pathological diagnosis [20]. However, in the clinical setting, a thicker needle makes the puncture procedure more difficult, particularly during transduodenal puncture in which the steep up-angle of the scope results in less flexibility and greater friction for the needle sheath. Moreover, this steep up-angle can distort the puncture needle, necessitating additional needles for a subsequent puncture. Given these facts, a needle that is flexible but can collect a sufficient volume of specimens for pathological assessment is needed for pancreatic core needle biopsy.

The EchoTip ProCore® needle (Wilson Cook Medical Inc., Winston-Salem, NC, USA) has a hollowed-out core trap on the side of the needle. This core trap system is designed to improve the acquisition of core biopsy specimens. Sterlacci et al. [21] reported that EUS tissue sampling using a 22-gauge (22G) needle with a core trap increased the accuracy of pathological diagnosis for abdominal masses compared with using a 22G aspiration needle. EchoTip ProCore® needles of various diameters are currently available. In Japan, the EchoTip ProCore 22G® (PC22) was approved for pancreatic EUS-FNA/B in March 2012 and the EchoTip ProCore 20G® needle (PC20) in December 2015. Notably, the PC20 has an anterograde, forward-bevel core trap, while the PC22 has a retrograde, reverse-bevel core trap. Additionally, the PC20 employs a new coil sheath resulting in greater flexibility of the needle, making procedures involving the duodenum easier. A recent study using porcine livers showed preferable tissue acquisition with the PC20 [22]. However, whether the PC20 can improve the diagnostic capability of EUS-FNA/B for pancreatic cancer in the real-world clinical setting has not been proven. In the present study, we retrospectively assessed the diagnostic efficacy and safety of the PC20 in patients with pancreatic cancer.

## Methods

### Patients

In this retrospective study, we investigated the clinical database of Kyorin University Hospital. We analyzed 191 patients who were diagnosed with pancreatic cancer and underwent their first EUS-FNA/B with the PC20, PC22, or Acquire 22G® (AC22) (Boston Scientific, Natick, MA, USA) for pancreatic solid lesions from April 2013 to October 2019. The final diagnosis of pancreatic cancer was confirmed by pathological diagnosis with EUS-FNA/B, percutaneous pancreatic biopsy, open biopsy, or ascites cytology. Some patients underwent EUS-FNA/B multiple times. Our board-certified pathologists at Kyorin University Hospital assessed both cytology and histology. The pathological diagnosis of each specimen was confirmed by multiple board-certified pathologists. A histological specimen from each pass was assessed. The diagnosis of pancreatic cancer was made when adenocarcinoma was histologically diagnosed in a specimen. In cytologic assessment, a specimen with class IV and V cells was diagnosed as pancreatic cancer. For specimens that could not be definitively diagnosed by these procedures, the diagnosis of pancreatic cancer was made based on the patient’s clinical course with scheduled imaging tests during the 12-month period following EUS-FNA/B.

### Ethics statement

The present study was approved by the Institutional Review Board of Kyorin University School of Medicine (IRB No. 1099) on 27 March 2018. This study was performed in accordance with the guidelines of the Declaration of Helsinki. Informed consent was obtained using an opt-out method.

### Procedure of EUS-FNA/B

All patients underwent conscious sedation by administration of midazolam (Maruishi Pharmaceutical, Osaka, Japan) and pentazocine (Maruishi Pharmaceutical). The EUS-FNA/B procedures were performed using a linear array echoendoscope (GF-UCT260®; Olympus Medical Systems, Tokyo, Japan or EG-580UT®; Fujifilm Medical, Tokyo, Japan) connected to a processor featuring a color Doppler function (EU-ME1, 2®; Olympus Medical Systems or SU-1®; Fujifilm Medical, respectively). EUS-FNA/B was performed by physicians with > 5 years of experience in EUS or by fellows in our department under the supervision of these experienced physicians. The target lesion for FNA/B was visually confirmed with EUS, and color Doppler was used to evaluate the vascularity along the puncture route. The stroke length is defined as the length of a tumor measured by EUS at the puncture line. The pancreatic solid tumor was punctured through the transgastric or transduodenal route with the PC20, PC22, or AC22. After the needle reached the target, the stylet was removed and suction was applied using a 10-ml syringe. For every puncture, 20 to-and-fro movements were employed. After removing the needle, the stylet was inserted and a sample was taken and placed on a glass slide. The filamentous sample was immersed in a 10% formalin solution for histological diagnosis, and the remaining specimen on the glass slide was smeared for cytological diagnosis. Following each pass, the endoscopists assessed the specimens by macroscopic on-site evaluation (MOSE). If a large amount of bleeding occurred at the first pass, a second pass was performed by changing the suction method to the slow-pull method. If the specimens were macroscopically insufficient, more passes were performed (maximum of six passes).

### MOSE

The result of MOSE was classified into three categories. In a filamentous sample of ≥ 5 mm, white tissue was defined as an adequate specimen and a reddish sample containing a large amount of blood as a clotted specimen. A sample of < 5 mm was defined as a fragment specimen.

### Accuracy of diagnosis of pancreatic cancer

Patients who were diagnosed with pancreatic cancer by the first EUS-FNA/B were defined as those who had obtained an “accurate diagnosis” by EUS-FNA/B. The accuracy of EUS-FNA/B was calculated as the proportion of patients who obtained an accurate diagnosis by EUS-FNA/B among all patients with pancreatic cancer.

### Statistical analysis

The chi-squared test and Fisher’s exact test were performed to compare patient backgrounds (sex, use of antiplatelet/anticoagulant agents, comorbid diabetes mellitus, smoking, gastric surgical history, location of pancreatic tumor, and puncture line), the accuracy of the pancreatic cancer diagnosis by EUS-FNA/B, the incidence of needle distortion, and the incidence of adverse events among the different core biopsy needles. For multiple comparisons of diagnostic accuracy, Bonferroni correction was employed to calculate the adjusted *p* value (*p-*adj). The Kruskal–Wallis test and Dunn’s test were performed to compare the patients’ age, maximum diameter of the tumor measured on contrast-enhanced computed tomography images, maximum stroke length for puncture measured on EUS images, puncture stroke length, and number of passes between needles. Statistical significance was defined as *p* < 0.05. GraphPad Prism version 8.1.2 (GraphPad Software, San Diego, CA, USA) was used for the statistical analyses.

## Results

### Patients’ backgrounds

We analyzed 191 patients who underwent their first EUS-FNA/B with the PC20, PC22, or AC22 from April 2013 to October 2019 at Kyorin University Hospital and were eventually diagnosed with pancreatic cancer. In Japan, the PC20, PC22, and AC22 were approved for EUS-FNA/B for pancreatic solid lesions by the Ministry of Health, Labour and Welfare in December 2015, March 2012, and October 2016, respectively. Among the 191 patients, the first EUS-FNA/B was performed in 53 patients from April 2013 to November 2015, in 13 patients from December 2015 to September 2016, and in 125 patients from October 2016 to October 2019. The PC20, PC22, and AC22 were used for 111, 68, and 12 patients, respectively. The patients’ characteristics are shown in Table 1. There was no significant difference in sex, age, tumor location, or puncture route among the needles. However, the maximum diameter of the tumor measured on contrast-enhanced computed tomography images was significantly different among the needles (PC20, 37.9 ± 1.2 mm; PC22, 32.9 ± 1.2 mm; AC22, 22.7 ± 2.6 mm; *p* < 0.0001). Although a sufficient stroke length of puncture was secured in all patients, the maximum stroke length for puncture measured on EUS images was shorter with the AC22 (22.1 ± 2.2 mm) (*p* < 0.01 and *p* < 0.01 compared with PC20 and PC22, respectively), while there was no significant difference between the PC20 and PC22 (30.6 ± 0.7 and 30.3 ± 0.8 mm, respectively; *p* > 0.99). The number of passes was significantly higher with the PC22 (4.1 ± 0.7) than PC20 (3.7 ± 0.9, *p* < 0.01) and AC22 (3.5 ± 0.6, *p* < 0.05).

**Table 1:** Patients characteristics

	PC20	PC22	AC22	*p* value
Number of patients	111	68	12	
Age (years) (median, range)	70 (40–87)	71 (47–85)	73 (52–80)	0.69^§^
Sex				0.35^#^
Male	59 (53.1%)	37 (54.4%)	9 (75%)	
Female	52 (46.9%)	31 (45.6%)	3 (25%)	
Anti-platelet/anti-coagulant agents	12 (10.8%)	11 (16.2%)	3 (25%)	0.29^#^
Diabetes Mellitus	32 (28.8%)	24 (21.6%)	2 (16.7%)	0.37^#^
Smoking	16 (14.4%)	8 (11.8%)	3 (25%)	0.47^#^
Post-operative stomach	1 (0.9%)	1 (1.47%)	0 (0%)	0.87^#^
Location of the pancreatic tumor				0.88^#^
Head	33 (29.7%)	19 (28.0%)	5 (41.7%)	
Body	56 (50.5%)	37 (54.4%)	5 (41.7%)	
Tail	22 (19.8%)	12 (17.6%)	2 (16.7%)	
Puncture line				0.71^#^
Trans gastric	85 (76.6%)	50 (73.5%)	8 (66.7%)	
Trans duodenal	26 (23.4%)	18 (26.5%)	4 (33.3%)	
Maximum diameter of tumor (mm) (mean ± SEM)	37.9 ± 1.2*^, $$$$^	32.9 ± 1.2^¶^	22.7 ± 2.6	< 0.0001^§^
Maximum stroke length of puncture (mm) (mean ± SEM)	30.6 ± 0.7^$$^	30.3 ± 0.8^¶¶^	22.1 ± 2.2	< 0.01^§^
Number of passes	3.7 ± 0.9**	4.1 ± 0.7^¶^	3.5 ± 0.6	< 0.01^§^

*PC20* EchoTip ProCore 20G, *PC22* EchoTip ProCore 22G, *AC22* Acquire 22G

^#^Chi-squared test among PC20, PC22, and AC22

^§^Kruskal–Wallis test among PC20, PC22, and AC22

^*^p < 0.05 and **p < 0.01, Dunn’s test; PC20 versus PC22

^$$^*p* < 0.01 and ^$$$$^*p* < 0.0001, Dunn’s test; PC20 versus AC22

^¶^*p* < 0.05 and ^¶¶^*p* < 0.01, Dunn’s test; PC22 versus AC22

### Diagnostic accuracy of EUS-FNA/B needles for pancreatic cancer

The diagnostic accuracy for pancreatic cancer with the PC20 was 96.4% (107/111), 81.1% (90/111), and 96.4% (107/111) by histology, cytology, and the combination of histology and cytology, respectively (Table 2). The accuracy with the PC22 was 58.8% (40/68), 63.2% (43/68), and 72.1% (49/68) and that with the AC22 was 75.0% (9/12), 83.3% (10/12), and 91.7% (11/12), respectively (Table 2). The diagnostic accuracy with the PC20 by histology, cytology, and the combination of histology and cytology was significantly better than that with the PC22 (*p-*adj < 0.0001, *p-*adj < 0.05, and *p-*adj < 0.0001, respectively), while there was no significant difference between the PC20 and AC22 (*p-*adj = 0.06, *p-*adj > 0.99, and *p-*adj > 0.99, respectively). The addition of cytological assessment to histological assessment improved the diagnostic accuracy with the PC22 and AC22 but not with the PC20. Comparison of diagnostic accuracy between two needles (PC20 vs. PC22, PC20 vs. AC22 and PC22 vs. AC22) are shown in Additional file 1: Table S1. Meanwhile, the diagnostic accuracy in each pancreatic location tended to be better in PC20 and AC22 compared to PC22 (Additional file 2: Table S2). Next, we examined the diagnostic efficacy of the first pass. The histological accuracy in the first pass was 85.6 (95/111) with the PC20, 33.8% (23/68) with the PC22, and 66.7% (8/12) with the AC22 (Table 3). The accuracy was significantly higher with the PC20 than PC22 (*p-*adj < 0.0001), while there was no significant difference between the PC20 and AC22 (*p-*adj = 0.32). The proportion of adequate specimens by MOSE among the samples from the first pass was 63.9% (71/111) with the PC20, 41.2% (28/68) with the PC22, and 50.0% (6/12) with the AC22. There was no significant difference among the needles (*p* = 0.20) (Table 3, Additional file 3: Table S3). The histological accuracy among adequate specimens and clotted specimens was 81.7% and 92.5% with the PC20, 39.3% and 30.0% with the PC22, and 83.3% and 50.0% with the AC22.

**Table 2:** Diagnostic accuracy for pancreatic cancer

	PC20	PC22	AC22	*p* value
Histology	96.4%^††††^ (107/111)	58.8% (40/68)	75.0% (9/12)	< 0.0001^#^
Cytology	81.1%^†^ (90/111)	63.2% (43/68)	83.3% (10/12)	< 0.05^#^
Combination of histology and cytology	96.4%^††††^ (107/111)	72.1% (49/68)	91.7% (11/12)	< 0.0001^#^

*PC20* EchoTip ProCore 20G, *PC22* EchoTip ProCore 22G, *AC22* Acquire 22G

^#^Chi-squared test among PC20, PC22, and AC22

^†^adjusted *p* < 0.05 and ^††††^adjusted *p* < 0.0001, Fisher’s exact test followed by Bonferroni correction; PC20 versus PC22

**Table 3:** Histological accuracy and adequacy in macroscopic on-site evaluation of specimens from 1st pass

	PC20	PC22	AC22	*p* value
Histological accuracy	85.6%^††††^ (95/111)	33.8% (23/68)	66.7% (8/12)	< 0.0001^#^
Macroscopic on-site evaluation	63.9% (71/111)	41.2% (28/68)	50.0% (6/12)	0.20^#^

*PC20* EchoTip ProCore 20G, *PC22* EchoTip ProCore 22G, *AC22* Acquire 22G

^#^Chi-squared test among PC20, PC22, and AC22

^††††^Adjusted *p* < 0.0001, Fisher’s exact test followed by Bonferroni correction; PC20 versus PC22

### Requirement of additional EUS-FNA/B needles

Among the 111 cases in which the PC20 was used, 5 (4.5%) required a biopsy needle change. The needle was distorted and the stylet could not be inserted in two of these five cases. In the other three cases, the needles were changed because of insufficient specimens (i.e., fragment specimens by MOSE). The PC22 was changed in 7 cases because of fragment specimens (10.3%, 7/68). Two cases in which the AC22 was used required a needle change because of fragment specimens (16.7%, 2/12). There was no significant difference in the requirement of a needle change among the different needle types (*p* = 0.20) (Table 4).

**Table 4:** The cases using additional needles

	Number of cases^#^	Reason of using additional needles
PC20	5 (4.5%; 5/111)	The needle got distorted and the stylet could not be inserted: 2 cases Insufficient specimens: 3 cases
PC22	7 (10.3%; 7/68)	Insufficient specimens: 7 cases
AC22	2 (16.7%; 2/12)	Insufficient specimens: 2 cases

*PC20* EchoTip ProCore 20G, *PC22* EchoTip ProCore 22G, *AC22* Acquire 22G

^#^*p* = 0.20, Chi-squared test among PC20, PC22, and AC22

### Adverse events related to EUS-FNA/B procedure

One patient in the PC20 group developed mild pancreatitis (0.9%, 1/111). The patient recovered with conservative treatment (fasting, fluid replacement, protease inhibitor, and antibiotic therapy). No adverse events occurred among the patients in the PC22 group (0.0%, 0/68) or AC22 group (0.0%, 0/12) (*p* = 0.54).

## Discussion

In this retrospective study, we examined the diagnostic efficacy of three puncture needles (PC20, PC22, and AC22) for the first EUS-FNA/B in patients with pancreatic cancer. The PC20 and PC22 employ a core trap system, which contributes to increased tissue acquisition and preservation of the histological structure [23]. A core trap can reportedly ensure effective cellularity for diagnosis [21, 24]. Acquiring tissue specimens with minimal structural damage increases the histopathological quality of the samples. High-quality specimens are crucial for assessment of the subtypes of malignancy (e.g., adenocarcinoma, adenosquamous carcinoma, anaplastic carcinoma, neuroendocrine carcinoma, and metastatic carcinoma) and performance of immunohistochemistry [25, 26]. Therefore, a core trap is an advantage in histopathological diagnosis of pancreatic tumors by EUS-FNA/B. However, although both the PC20 and PC22 have a core trap, the present study demonstrated that diagnostic accuracy was significantly higher with the PC20 than PC22. Two possible factors led to the difference in diagnostic accuracy between the PC20 and PC22: (1) the difference in the needle gauge and (2) the difference in the design of the core trap. A thicker needle is reportedly more useful for collection of core tissue [27] and preservation of the histological structure of specimens [18] than is a thinner needle. Meanwhile, Armellini et al. [28] reported that the forward-bevel core trap (PC20) enables the acquisition of more tissue than the reverse-bevel core trap (PC22). This is thought to be because the forward-bevel core trap catches the tissue while the needle moves forward [28]. Additionally, the core trap is larger in the PC20 than PC22 (2.9 vs. 2.0 mm, respectively). However, there is little difference in the distance from the tip to the core trap between the PC20 and PC22 (3.8 vs. 3.9 mm, respectively). This suggests that the stroke length measured by EUS imaging does not drive the needle choice between the PC20 and PC22. Our findings suggest that the PC20 is preferable for the histological diagnosis. The total accuracy (combined histology and cytology) with the PC20 (96.4%) was equal to the histological result; i.e., there was no additional diagnostic impact of cytology. Additionally, even in samples defined as clotted specimens by MOSE, the histological accuracy of clotted specimens was 92.5% with the PC20. In one study, the histological sensitivity was 92.4% in whitish tissue and 40.8% in blood clots [29]. Although a sample obtained by a thicker needle is contaminated by more blood [30], our results suggest that clotted specimens obtained by the PC20 can still be used for histological assessment.

We also assessed the diagnostic accuracy of the AC22 in this study. The AC22 is a relatively new needle with a diagnostic accuracy of 94.0–96.7% [31–33]. Ishikawa et al. [34] reported that the AC22 was better than a conventional needle in acquiring cells. The AC22 is designed to hold and cut the tissue with multiple sharp structures on the tip (fork tip). This characteristic structure contributes to better sample collection even in a lesion with dense fibrotic change, different from most conventional EUS-FNA/B needles [32, 35]. A randomized trial by Bang et al. [36]. demonstrated that AC22 showed better diagnostic accuracy for pancreatic masses compared to 22-gauge conventional needles and PC22 In an animal experiment, there was no significant difference in the core tissue acquisition ability between the PC20 and AC22 [22]. Considering that the AC22 is thinner (22G) than the PC20 (20G), the fork-tip structure of the AC22 could be more advantageous for tissue collection than the core trap of the PC20. However, a recent study proposed that the side bevel, which moves to and fro in the tumor during the sampling procedure, can cut the tumor surface more effectively than the needle tip within the tumor [37]. Thus, considering the tip structure of the AC22, this needle might be more effective than other types particularly for cases in which a long puncture stroke length cannot be secured. Fujita et al. [38] compared the diagnostic accuracy between the AC22 and a conventional needle and found no difference between the two needles for > 20-mm tumors; however, there was a significant difference for < 19-mm tumors. There was no significant difference in the histological accuracy between the PC20 and AC22 in the present study (*p* = 0.02, *p*-adj = 0.06). A sufficient stroke length was secured for all patients; however, given that the puncture stroke length was significantly shorter with the AC22 than PC20, it is difficult to draw a strict conclusion regarding the efficacy of the PC20 versus AC22 from this study. However, our findings suggest that as long as a sufficient stroke length can be secured, the diagnostic accuracy of both the PC20 and AC22 is sufficiently high. Given our results that the histological accuracy tended to be higher with the PC20 than AC22 as well as the small number of patients in whom the AC22 was used, we speculate that the PC20 is suitable for cases in which histological assessment including immunohistochemistry can be critical for diagnosis, while the AC22 is suitable for small tumors in which it is difficult to secure a long stroke length.

In EUS-FNA/B, transduodenal puncture is challenging in some cases because of the steep up-angle of the scope [23, 39]. A thicker needle with less flexibility has a disadvantage in this procedure, and endoscopists need to use additional needles in some cases. In this study, however, the operability of the PC20 was not different from that of the PC22 and AC22. This finding suggests that the PC20 can be used for transduodenal puncture similarly to 22G needles. In terms of adverse events, one case of mild pancreatitis was observed in the PC20 group (0.9%), and no adverse events occurred in the PC22 and AC22 groups. No serious complications such as active bleeding or perforation occurred with any needles. A systematic review on the safety of EUS-FNA revealed a complication rate of 0.98% and mortality rate of 0.02% [40]. The needle diameter is not thought to be associated with the adverse event rate [41, 42]. Considering the results of both our study and previous studies, it seems that there is no considerable difference in safety among the PC20, PC22, and AC22.

This study had several limitations. First, because it was a single-center, retrospective study, the EUS-FNA/B skills might have varied over time and/or among endoscopists, and the needle selection by each operator might have been biased. There could be a possibility that the characteristic structure of AC22, the multiple-sharp-structure tip, impacts the needle selection. Second, the AC22 was approved for EUS-FNA/B for pancreatic solid lesions in October 2016 in Japan, and in our facility, the AC22 has been used in much fewer patients than the PC20 and PC22. It is needed to perform a prospective study with randomizing the needle selection and setting the specific criteria for the tumor size and the number of passes. Additionally, in this study, the stroke length of puncture under EUS images was not controlled between the PC20 and AC22. A study in which the stroke length is identical between the needles is necessary for direct comparison of the efficacy of these needles.

## Conclusions

In conclusion, this study demonstrated that the PC20 has better diagnostic efficacy than the PC22. Although further study is needed to compare the PC20 and AC22, the high histological accuracy of the PC20 could be advantageous for lesions in which histological assessment is critical, while the AC22 could be a good option particularly for small lesions. Evidence-based needle selection could contribute to the improvement of diagnosing pancreatic cancer.

## Supplementary Information

**Additional file 1.** **Table S1.** Comparison of diagnostic accuracy between two needles.

**Additional file 2.** **Table S2.** Comparison of diagnostic accuracy in each pancreatic location between two needles.

**Additional file 3.** **Table S3.** Comparison of macroscopic on-site evaluation between two needles.

## Data Availability

The datasets used and analyzed during the current study are available from the corresponding author on reasonable request.

## Abbreviations

AC22: Acquire 22G®
EUS-FNA/B: Endoscopic ultrasound-guided fine-needle aspiration/biopsy
MOSE: Macroscopic on-site evaluation
PC20: EchoTip ProCore 20G®
PC22: EchoTip ProCore 22G®
