# Supplementary material for: Effectiveness of introducing a 20-gauge core biopsy needle with a core trap in EUS-FNA/B for diagnosing pancreatic cancer
Source: BMC Gastroenterol. 2021 Jan 6;21:8. doi: 10.1186/s12876-020-01583-7 (PMC7789690; doi:10.1186/s12876-020-01583-7)
Supplement: Supplementary file 1 — Additional file 1. Table S1. Comparison of diagnostic accuracy between two needles. [file 12876_2020_1583_MOESM1_ESM.docx]

Table S1. Comparison of diagnostic accuracy between two needles.

|  | PC20 vs. PC22 | PC20 vs. AC22 | PC22 vs. AC22 |
| --- | --- | --- | --- |
| Histology | 96.4% vs. 58.8%  *p* < 0.00010  (*p*-adj < 0.00030) | 96.4% vs. 75.0%  *p* = 0.020  (*p*-adj = 0.061) | 58.8% vs. 75.0%  *p* = 0.35  (*p*-adj > 0.99) |
| Cytology | 81.1% vs. 63.2%  *p* = 0.013  (*p*-adj = 0.039) | 81.1% vs. 83.3%  *p* = 1.0  (*p*-adj > 0.99) | 63.2% vs. 83.3%  *p* = 0.32  (*p*-adj = 0.96) |
| Combination of Histology and Cytology | 96.4% vs. 72.1%  *p* < 0.00010  (*p*-adj < 0.00030) | 96.4% vs. 91.7%  *p* = 0.41  (*p*-adj > 0.99) | 72.1% vs. 91.7%  *p* = 0.28  (*p*-adj = 0.84) |

*p*-value: Fisher’s exact test between two groups.

*p*-adj: Adjusted *p* value with Bonferroni correction among three groups (PC20, PC22 and AC22).
