# Supplementary material for: Effectiveness of introducing a 20-gauge core biopsy needle with a core trap in EUS-FNA/B for diagnosing pancreatic cancer
Source: BMC Gastroenterol. 2021 Jan 6;21:8. doi: 10.1186/s12876-020-01583-7 (PMC7789690; doi:10.1186/s12876-020-01583-7)
Supplement: Supplementary file 2 — Additional file 2. Table S2. Comparison of diagnostic accuracy in each pancreatic location between two needles. [file 12876_2020_1583_MOESM2_ESM.docx]

Table S2. Comparisons of diagnostic accuracy in each pancreatic location between two needles.

|  | PC20 vs. PC22 | PC20 vs. AC22 | PC22 vs. AC22 |
| --- | --- | --- | --- |
| Histology | | | |
| Head | 93.9% vs. 47.4%  *p* = 0.00020  (*p*-adj = 0.00060) | 93.9% vs. 80.0%  *p* = 0.35  (*p*-adj > 0.99) | 47.4% vs. 80.0%  *p* = 0.33  (*p*-adj = 0.98) |
| Body | 98.2% vs. 62.2%  *p* = 0.00010  (*p*-adj = 0.00030) | 98.2% vs. 80.0%  *p* = 0.16  (*p*-adj = 0.48) | 62.2% vs. 80.0%  *p* = 0.64  (*p*-adj > 0.99) |
| Tail | 95.5% vs. 66.7%  *p* = 0.042  (*p*-adj = 0.13) | 95.5% vs. 50%  *p* = 0.16  (*p*-adj = 0.49) | 66.7% vs. 50.0%  *p* > 0.99  (*p*-adj > 0.99) |
| Cytology | | | |
| Head | 69.7% vs. 47.4%  *p* = 0.00020  (*p*-adj = 0.00060) | 69.7% vs. 80.0%  *p* > 0.99  (*p*-adj > 0.99) | 47.4% vs. 80.0%  *p* = 0.053  (*p*-adj = 0.16) |
| Body | 87.5% vs. 75.7%  *p* = 0.00092  (*p*-adj = 0.0027) | 87.5% vs. 80.0%  *p* = 0.16  (*p*-adj = 0.48) | 75.7% vs. 80.0%  *p* > 0.99  (*p*-adj > 0.99) |
| Tail | 81.8% vs. 50.0%  *p* = 0.11  (*p*-adj = 0.34) | 81.8% vs. 100%  *p* = 0.38  (*p*-adj > 0.99) | 50.0% vs. 100%  *p* > 0.99  (*p*-adj > 0.99) |
| Combination of Histology and Cytology | | | |
| Head | 96.9% vs. 57.9%  *p* = 0.0027  (*p*-adj = 0.0081) | 96.9% vs. 100%  *p* > 0.99  (*p*-adj > 0.99) | 57.9% vs. 100%  *p* = 0.13  (*p*-adj = 0.39) |
| Body | 96.4% vs. 81.1%  *p* = 0.0060  (*p*-adj = 0.018) | 96.4% vs. 80.0%  *p* > 0.99  (*p*-adj > 0.99) | 81.1% vs. 80.0%  *p* = 0.57  (*p*-adj > 0.99) |
| Tail | 95.5% vs. 66.7%  *p* = 0.042  (*p*-adj = 0.13) | 95.5% vs. 100%  *p* = 0.16  (*p*-adj = 0.49) | 66.7% vs. 100%  *p* > 0.99  (*p*-adj > 0.99) |

*p*-value: Fisher’s exact test between two groups.

*p*-adj: Adjusted *p* value with Bonferroni correction among three groups (PC20, PC22 and AC22).
