# Supplementary material for: Effectiveness of introducing a 20-gauge core biopsy needle with a core trap in EUS-FNA/B for diagnosing pancreatic cancer
Source: BMC Gastroenterol. 2021 Jan 6;21:8. doi: 10.1186/s12876-020-01583-7 (PMC7789690; doi:10.1186/s12876-020-01583-7)
Supplement: Supplementary file 3 — Additional file 3. Table S3. Comparison of macroscopic on-site evaluation between two needles. [file 12876_2020_1583_MOESM3_ESM.docx]

Table S3. Comparison of macroscopic on-site evaluation between two needles.

|  | PC20 vs. PC22 | PC20 vs. AC22 | PC22 vs. AC22 |
| --- | --- | --- | --- |
| Macroscopic on-site evaluation | 63.9% vs. 41.2%  *p* = 0.0034  (*p*-adj = 0.010) | 63.9% vs. 50.0%  *p* = 0.36  (*p*-adj > 0.99) | 41.2% vs. 50.0%  *p* = 0.75  (*p*-adj > 0.99) |

*p*-value: Fisher’s exact test between two groups.

*p*-adj: Adjusted *p* value with Bonferroni correction among three groups (PC20, PC22 and AC22).
